# Prometastatic Effect of ATX Derived from Alveolar Type II Pneumocytes and B16-F10 Melanoma Cells

**DOI:** 10.3390/cancers14061586

**Published:** 2022-03-21

**Authors:** Mélanie A. Dacheux, Sue Chin Lee, Yoojin Shin, Derek D. Norman, Kuan-Hung Lin, Shuyu E, Junming Yue, Zoltán Benyó, Gábor J. Tigyi

**Affiliations:** 1 Department of Physiology, College of Medicine, University of Tennessee Health Science Center (UTHSC), Memphis, TN 38163, USA; mdacheux@uthsc.edu (M.A.D.); slee84@uthsc.edu (S.C.L.); yshin2@uthsc.edu (Y.S.); dnorman7@uthsc.edu (D.D.N.); klin10@uthsc.edu (K.-H.L.); 2 Department of Pathology and Laboratory Medicine, College of Medicine, University of Tennessee Health Science Center (UTHSC), Memphis, TN 38163, USA; se1@uthsc.edu (S.E.); jyue@uthsc.edu (J.Y.); 3 Institute of Translational Medicine, Semmelweis University, H-1428 Budapest, Hungary; benyo.zoltan@med.semmelweis-univ.hu

**Keywords:** autotaxin, lysophosphatidic acid, metastasis, B16-F10, melanoma, alveolar type II cells, tumor microenvironment, tumor immunity

## Abstract

**Simple Summary:**

Only a few studies have reported a role of alveolar type II epithelial (ATII) pneumocytes in the development of the lung metastatic niche. In the present study, we investigated the contribution of autotaxin (ATX) produced by ATII cells in driving the progression of B16-F10 melanoma-derived lung metastases. We found that the metastatic burden is reduced when the ATX gene is deleted from both ATII and B16-F10 cells. We detected increased levels of cytokines such as IFNγ and TNFα, which could favor the increase in infiltrating CD8^+^ T cells observed in the tumor regions. Our findings suggest that a concomitant inhibition of ATX from both stromal and cancer cells may, in part, modulate the antitumor response to better control metastatic progression.

**Abstract:**

Although metastases are the principal cause of cancer-related deaths, the molecular aspects of the role of stromal cells in the establishment of the metastatic niche remain poorly understood. One of the most prevalent sites for cancer metastasis is the lungs. According to recent research, lung stromal cells such as bronchial epithelial cells and resident macrophages secrete autotaxin (ATX), an enzyme with lysophospholipase D activity that promotes cancer progression. In fact, several studies have shown that many cell types in the lung stroma could provide a rich source of ATX in diseases. In the present study, we sought to determine whether ATX derived from alveolar type II epithelial (ATII) pneumocytes could modulate the progression of lung metastasis, which has not been evaluated previously. To accomplish this, we used the B16-F10 syngeneic melanoma model, which readily metastasizes to the lungs when injected intravenously. Because B16-F10 cells express high levels of ATX, we used the CRISPR-Cas9 technology to knock out the ATX gene in B16-F10 cells, eliminating the contribution of tumor-derived ATX in lung metastasis. Next, we used the inducible Cre/loxP system (Sftpc-CreER^T2^/Enpp2^fl/fl^) to generate conditional knockout (KO) mice in which ATX is specifically deleted in ATII cells (i.e., Sftpc-KO). Injection of ATX-KO B16-F10 cells into Sftpc-KO or Sftpc-WT control littermates allowed us to investigate the specific contribution of ATII-derived ATX in lung metastasis. We found that targeted KO of ATX in ATII cells significantly reduced the metastatic burden of ATX-KO B16-F10 cells by 30% (unpaired *t*-test, *p* = 0.028) compared to Sftpc-WT control mice, suggesting that ATX derived from ATII cells could affect the metastatic progression. We detected upregulated levels of cytokines such as IFNγ (unpaired *t*-test, *p* < 0.0001) and TNFα (unpaired *t*-test, *p* = 0.0003), which could favor the increase in infiltrating CD8^+^ T cells observed in the tumor regions of Sftpc-KO mice. Taken together, our results highlight the contribution of host ATII cells as a stromal source of ATX in the progression of melanoma lung metastasis.

## 1. Introduction

The most common cancers in the United States are skin cancers, among which melanoma is the deadliest form. Metastatic melanoma has a poor prognosis with a five-year survival rate of 27%. Although targeted treatments and immune checkpoint therapies have improved the long-term survival rate of patients with metastatic melanoma, the mechanisms governing metastasis and treatment failure remain poorly understood [1,2].

During the metastatic process, select cancer cells disseminate from a primary lesion and migrate to a distant tissue. In this mechanism, these cancer cells need to successfully invade the basement membrane, extravasate the blood vessel, escape immunosurveillance, seed at a secondary site, and promote the development of a metastatic niche that favors their growth [3]. The development of the metastatic niche is influenced by the tumor microenvironment (TME), where complex interactions occur between cancer cells and the surrounding tissue components [4]. Increasing evidence suggests that the TME forms a critical barrier to successful treatment [5,6,7]. Autotaxin (ATX), also known as ectonucleotide pyrophosphatase/phosphodiesterase 2 (ENPP2), generates lysophosphatidic acid (LPA), a growth-factor-like lipid mediator that serves as a ligand for six G-protein-coupled receptors (LPAR; LPAR_1_-_6_). This signaling pathway regulates TME biology [8,9]. ATX was first identified in melanoma cells as a motility-promoting factor [10]. The level of ATX is upregulated in many types of cancers, and the ATX–LPA–LPAR pathway promotes cancer cell proliferation [11], migration, survival, invasion, angiogenesis, metastasis, and therapeutic resistance [8,12,13], in both humans and mice.

Although some cancer cell lines secrete high levels of ATX protein, recent investigations have turned to focus on the role of stromal-derived ATX in tumorigenesis and metastasis. For example, in breast cancer, mammary adipose tissue and cancer-associated fibroblasts secrete ATX, which in turn drives breast cancer progression [11]. Moreover, ATX secreted by platelets promotes breast cancer colonization to the bone [14]. In pancreatic cancer, pancreatic stellate cells promote pancreatic ductal adenocarcinoma progression through the release of lysophosphatidylcholine (LPC), which is hydrolyzed into LPA by ATX [15]. Conditional genetic deletion of ATX from bronchial epithelial cells and macrophages diminishes neoplastic lesions in a urethane-induced lung cancer mouse model. Moreover, ATX expression is increased in the lungs of patients with lung cancer and has been linked to the pathogenesis of lung cancer [16]. In the C57BL/6-B16-F10 syngeneic murine melanoma lung metastasis model, we previously demonstrated that host LPAR_1_ and LPAR_5_ promote the seeding of metastases and that ATX produced by cancer cells enhances melanoma cell migration and invasion [17,18].

Lungs represent one of the most common sites for metastasis of multiple types of cancers [19]. Although ATII cells represent approximately 5% of the alveolar surface area, they play an essential role as the precursors of type I pneumocytes [20] and in maintaining pulmonary tissue homeostasis via the coordination of the host defense response and the secretion of lipid and protein surfactants [21]. In a lung injury model, endogenous ATX is secreted by alveolar epithelial cells in vivo and by the adenocarcinoma A549 cell line and primary human small airway epithelial cells in vitro [22]. In the lung tissue of patients with idiopathic pulmonary fibrosis, increased ATX expression was detected in both bronchiolar and alveolar epithelium, alveolar macrophages, and fibroblastoid cells [23]. These studies highlight the hypothesis that many cell types of lung stroma could provide a rich source of ATX in diseases.

Although we showed previously that rat alveolar epithelial type II cells (ATII) express ATX [18], the contribution of this source of ATX has not yet been investigated in the context of lung metastasis. Therefore, we set out to determine the contribution of ATII-derived ATX in B16-F10 metastasis by crossing Sftpc-CreER^T2^ mice with Enpp2^fl/fl^ mice to generate conditional knockout (KO) mice in which ATX is specifically deleted in ATII cells (i.e., Sftpc-KO). To eliminate the contribution of tumor-derived ATX in the metastatic process, we injected B16-F10 tumor cells lacking ATX (ATX-KO B16-F10) into Sftpc-KO mice and Sftpc-WT control littermates. We found that deletion of ATX in ATII cells reduced the number of metastatic lung nodules of ATX-KO B16-F10 cells by 30% compared to Sftpc-WT control mice, suggesting that ATX derived from ATII cells could affect the metastatic progression. We also found that the levels of IFNγ and TNFα cytokines and the infiltration by CD8^+^ cytotoxic T lymphocytes show characteristic changes in B16-F10 lung metastasis.

## 2. Materials and Methods

### 2.1. CRISPR-Cas9-Edited B16-F10 Cells and Isolation of Murine ATII Cells 

B16-F10 melanoma cells (ATCC; CRL-6475) were used to generate a pool of wild-type (WT) and CRISPR-Cas9-edited ATX-knockout (KO) B16-F10 cells by Synthego Inc. (Menlo Park, CA, USA). Briefly, the single guide RNA (sgRNA) sequence 5′-UCUCCAUGGACCAACACAUC-3′ was used to create frameshift mutations in the coding region of *Enpp2* (*Atx*) exon 3, chromosome 15. To generate ATX-KO B16-F10 cells, ribonucleoproteins containing Cas9 endonuclease and sgRNA were electroporated into these cells; to generate WT cells, the electroporation was performed without sgRNA. The end products were two mixed populations, a CRISPR-Cas9-edited ATX-KO pool (ATX-KO B16-F10) and a control WT pool (WT B16-F10), respectively. The editing efficiency was analyzed through Synthego Performance Analysis using the ICE analysis program (2019.v2.0. Synthego). WT and ATX-KO B16-F10 cells were cultured in Gibco Minimum Essential Media (MEM; Thermo Fisher Scientific, Pittsburgh, PA, USA), supplemented with 5% heat-inactivated Hyclone FBS (Thermo Fisher Scientific), 2 mM L-glutamine, 1X MEM vitamins (Gibco, 11120-053), 1X MEM non-essential amino acids (Gibco, 11140-050), and 1 mM sodium pyruvate (Gibco, 11360-070). To verify the genotypes by PCR amplification, we used Enpp2 primers: (F) 5′-GTGTCACTGAGCCTTTTGCT-3′ and (R) 5′-CTATACTGACATGCATTTCACAC-3′.

Primary mouse ATII cells were isolated according to published protocols [24,25] with minor modifications. Briefly, mice were anesthetized with a cocktail of ketamine and xylazine (Zoetis Inc., Parsippany, NJ, USA and Covetrus Inc., Portland, ME, USA respectively), and the hearts were perfused with 10 mL of sterile PBS. The lungs were inflated with 2 mL dispase solution (Corning, Inc., Corning, NY, USA) followed by 0.5 mL of 1% low melting point agarose (Sigma-Aldrich, Burlington, MA, USA). Lungs were removed and digested in 3 mL dispase solution with gentle agitation at room temperature for 45 min. Cells were dissociated mechanically in DMEM dissociation medium, consisting of DMEM (Thermo Fisher Scientific, Waltham, MA, USA) supplemented with 10% heat-inactivated FBS, 2 mM L-glutamine, 1% penicillin–streptomycin, 10 μg/mL gentamicin (Thermo Fisher Scientific), 2.5 μg/mL amphotericin (Sigma-Aldrich), and 0.1 mg/mL DNase I (Qiagen, Venlo, The Netherlands). The supernatant was sequentially filtered through 70 μm, 40 μm (Falcon Inc., Daytona Beach, FL, USA), and 30 μm mesh filters (MACS Miltenyi Biotec, Bergisch Gladbach, Germany). The cell suspension was plated for 3 h on a cell culture dish that was coated with biotinylated anti-mouse anti-CD45, anti-Ter119, anti-CD16/32, and anti-CD31 antibodies (all from Becton-Dickinson, Franklin Lakes, NJ, USA), and subsequently cultured in mouse ATII growth medium consisting of DMEM supplemented with 10% heat-inactivated FBS, 10 mM HEPES, 4 mM L-glutamine, 1% penicillin–streptomycin, 10 μg/mL Primocin (InvivoGen), and 2.5 μg/mL amphotericin. The resulting ATII cells were plated in 35 mm wells coated with fibronectin (Sigma-Aldrich) and laminin (Corning) and cultured up to 7 days in mouse ATII growth medium that was replaced daily.

### 2.2. Animal Models

All animal procedures were approved by the University of Tennessee Health Science Center (UTHSC) Institutional Animal Care and Use Committee (IACUC) and were in accordance with the Guide for the Care and Use of Laboratory Animals.

C57BL/6J and B6.129S-Sftpc^tm1(cre/ERT2)Blh^/J (Sftpc-CreER^T2^) mice were obtained from The Jackson Laboratories (Bar Harbor, ME, USA). Sftpc-CreER^T2^ mice express a tamoxifen-inducible Cre recombinase, where the Cre activity is specifically expressed in ATII cells. Enpp2 floxed mice (Enpp2^fl/fl^) that possess loxP sites flanking exons 6 and 7 of *Enpp2* [26] were a gift from Dr. Wouter H. Moolenaar (The Netherlands Cancer Institute, Amsterdam, Netherlands). To generate conditional ATX-KO ATII mice, we crossed Sftpc-CreER^T2^ and Enpp2^fl/fl^ mice. Genotypes used for the experiments were (Sftpc-Cre^+^, Enpp2^fl/fl^), abbreviated as Sftpc-KO, for the ATX-deleted ATII mice, and (Sftpc-Cre^+^, Enpp2^WT^), abbreviated as Sftpc-WT, for the WT littermates. Genotyping of mice was performed by PCR amplification on tail genomic DNA using Platinum Direct PCR Universal Master Mix (Invitrogen, San Diego, CA, USA), according to the manufacturer’s protocol. The following primers were used: Sftpc-common allele: (F) 5′-TGCTTCACAGGGTCGGTAG-3′; Sftpc-mutant allele: (R) 5′-ACACCGGCCTTATTCCAAG-3′; Sftpc-WT allele: (R) 5′-CATTACCTGGGGTAGGACCA-3′; Enpp2-common allele: (R) 5′-ACAGACTTCTCTGAAGCTGAC-3′; Enpp2-flox allele: (F) 5′-GCACATACCTTTAATTCCAGCAC-3′; Enpp2-WT allele: (F) 5′-CATTTCCATTCCCTGCTCC-3′.

In the metastasis model, WT or CRISPR-Cas9-edited ATX-KO B16-F10 cells (1 × 10^5^) were inoculated into WT C57BL/6J mice via the tail vein. For the experiments investigating the contribution of ATX-producing ATII cells, 1.5 × 10^5^ cells were inoculated into the tail vein of either Sftpc-WT or Sftpc-KO mice. All mice were euthanized using an overdose of inhaled isoflurane (Pivetal, Henry Schein) on post-inoculation day 21. Terminal blood collection was performed in heparin-coated tubes (Sarstedt) and EDTA-coated tubes, and plasma was rapidly isolated and stored at −80 °C. Bronchoalveolar lavage fluid (BALF) was collected using 2 × 600 μL of PBS. Subsequently, lungs were inflated with 10% buffered formalin (Thermo-Fisher), the metastatic nodules on the lung surface were counted, and the excised lungs were stored in 10% buffered formalin. Necropsy was also performed to identify the presence of extrapulmonary metastases.

### 2.3. Tamoxifen Treatment

To induce the deletion of ATX in ATII cells, Sftpc-KO mice were administered daily intraperitoneal (IP) injections of 100 mg/kg/day tamoxifen (TAM, Sigma-Aldrich) diluted in absolute ethanol and corn oil (Sigma-Aldrich; *v*/*v* 1:9) for 5 days. The Sftpc-WT control mice were treated the same way.

### 2.4. Measurement of ATX Activity

ATX activity was measured in conditioned media (CM) of WT and ATX-KO B16-F10 cells or BALF from mice injected with WT or ATX-KO B16-F10 cells, as described in [18]. Briefly, the fluorogenic LPC analog, FS-3 (Echelon Biosciences, Salt Lake City, UT, USA) was used as a substrate for ATX. For measurement of ATX activity in culture medium, CM was collected after 18 h of incubation in serum-free medium, clarified by centrifugation, filtered through a 0.22-μm filter, and concentrated using an Amicon Ultra 30,000 Centrifugal Filter Unit (Millipore; Billerica, MA, USA). For measurement of the activity in biological fluids, BALF was rapidly stored at −80 °C after collection and thawed on ice just before the assay. Five hundred microliters of BALF was then concentrated to 150 μL using an Amicon Ultra 10,000 Centrifugal Filter Unit (Millipore; Billerica, MA, USA). Activity was measured by incubating 30 μL of cell CM or murine BALF with 2 μM FS-3 substrate and 10 μM BSA (Sigma-Aldrich) in assay buffer consisting of 50 mM Tris, (pH = 8.0), 140 mM NaCl, 5 mM KCl, 1 mM CaCl_2_, and 1 mM MgCl_2_. A 10 nM final concentration of purified recombinant human ATX was used as a positive control. Fluorescence was read every 2 min for 4 h at 37 °C at wavelengths of 485 nm and 538 nm using a FlexStation3 plate reader (Molecular Devices, San Jose, CA, USA).

### 2.5. Western Blotting

We verified the presence of ATX protein in cell lysates using Western blotting (WB). WT and ATX-KO B16-F10 cells or ATII cells were plated to confluency (2 × 10^6^ cells/T75 flask or 5 × 10^5^ cells/well in a 6-well plate, respectively) in their respective serum-free medium for 18 h at 37 °C. Cells were washed twice with ice-cold PBS and lysed with M-PER Mammalian Protein Extraction Reagent (Thermo-Fisher) supplemented with 1% protease inhibitor cocktail (Sigma-Aldrich). Protein concentrations were estimated using a BCA kit (Thermo Fisher Scientific). A total of 100 μg (B16-F10 cells) or 150 μg protein (ATII cells) per lane was separated on 8% SDS-PAGE gels and transferred to a nitrocellulose membrane. The membrane was blocked with 5% non-fat milk in TBST (Bio-Rad) for 2 h and incubated overnight at 4 °C with a 1:1000 dilution of primary rat anti-4F1 ATX antibodies (gift from Dr. Junken Aoki, University of Tokyo). The membrane was rinsed and incubated with a 1:2000 dilution of HRP-conjugated goat anti-rat secondary antibodies (Thermo-Fisher) for 1 h at room temperature. ATX was detected using enhanced chemiluminescence (ECL) solution from a Super Signal West Pico Chemiluminescent Substrate kit (Thermo-Fisher) for B16-F10 cells or a Super Signal West Atto Maximum Sensitivity Substrate kit (Thermo-Fisher) for ATII cells and visualized using a Bio-Rad Gel Imager. Detected ATX signals were normalized to actin as a loading control, as follows: The membrane was subsequently stripped of antibodies with Restore Western Blot Stripping Buffer (Thermo-Fisher) according to the manufacturer’s protocol and incubated with mouse anti-actin primary antibody (Chemicon, 1:600,000 dilution) overnight at 4 °C. Secondary goat anti-mouse HPR antibody (Sigma-Aldrich) was used at a dilution of 1:5000 and incubated for 1 h at room temperature.

For ATII cells, a TNFα-stimulation step was added prior to the 18 h incubation. To do so, 10 ng/mL of recombinant mouse TNFα (Biolegend, San Diego, CA, USA) was added to the serum-free medium to stimulate ATX production.

### 2.6. Histology and Immunostaining

Excised lungs were paraffinized and cut into 5 μm sections. Sections were stained with a Hematoxylin & Eosin Stain kit (Vector Laboratories, Burlingame, CA, USA) according to the manufacturer’s protocol. Immunohistology was used to identify the spatial distribution of CD4^+^ and CD8^+^ T cells or CD68^+^ macrophage cells. After deparaffinization and rehydration, antigen retrieval was performed for 20 min at sub-boiling temperature with 10 mM of citrate buffer (pH = 6.0). Immunostaining was performed using VectaStain Elite ABC and Avidin/Biotin Blocking Kits, biotinylated anti-rat IgG, and ImmPACT DAB kit (all from Vector Laboratories), according to the manufacturers’ instructions. The dilutions of the primary antibodies used were as follows: CD68 (DAKO) 1:200, CD4 (Roche, Basel, Switzerland) 1:200, and CD8 (Invitrogen) 1:100. Sections were counterstained with hematoxylin.

### 2.7. Immunofluorescence

B16-F10 cells were cultured in a chamber slide system, washed twice with ice-cold PBS, and fixed in ice-cold methanol for 5 min. Slides were washed three times in PBS (5 min/wash), blocked using PBS containing 3% serum for 1 h at room temperature, and washed three times in PBS. Slides were incubated with anti-4F1 ATX primary antibodies (1:100 in PBS containing 0.1% BSA) overnight at 4 °C. Slides were washed three times in PBS and incubated in a dark chamber with AlexaFluor488-conjugated anti-rat secondary antibodies (Abcam; 1:1000 in PBS containing 0.1% BSA) for 1 h at room temperature. Slides were washed three times in PBS and counter-stained with Hoechst (Life Technologies; 1:5000 in PBS) for 5 min at room temperature in a dark chamber and washed again three times in PBS. Coverslips were affixed to slides with mounting medium. Slides were visualized using a Nikon Eclipse Ti microscope.

### 2.8. Mass Spectrometry

All lysophospholipids (16:0-LPA, 17:0-LPA, 18:0-LPA, 18:1-LPA, 18:2-LPA, and 20:4-LPA) were purchased from Avanti Polar lipids, Inc. Blood samples were collected in EDTA-coated tubes, and plasma samples were isolated and stored at −80 °C. Where indicated, blood was allowed to clot for serum collection and stored at −80 °C. Fifty microliters of plasma or serum was mixed with nine volumes of acidic methanol, homogenized for 3 min in an ultrasonic bath, and centrifuged at 14,000× *g* for 10 min at 4 °C. The supernatant was separated, dried, and resuspended in 100 μL of methanol. Ten microliters was injected into the AB Sciex Qtrap 4500 LC-MS/MS for analysis according to [27], with minor modifications. Briefly, LC separation was performed with a Capcell Park ACR column (Shiseido, Tokyo, Japan) with a gradient of elution of solvent A (5 mM ammonium formate in water) and solvent B (5 mM of ammonium formate in acetonitrile). pH of both solvents was adjusted to 4 using formic acid. The elution program was as follows: Starting condition was set at 55% of solvent B for 10 min. Then, from 10 to 40 min, a linear gradient to 90% of solvent B was applied and held for 7 min. Finally, the mobile phase was reset to 55% of solvent B for 3 min.

### 2.9. Transwell Cell Migration Assay

Cell transmigration assays were performed using the 24-well CellQart transwell inserts with 8 µm pore size (Sterlitech Corporation, Auburn, WA, USA) that were coated with fibronectin for 2 h at 37 °C prior to use. Fibronectin solution was then removed and 2 × 10^5^ cells were plated per insert and incubated for 1 h at 37 °C in 5% CO_2._ The lower chambers were filled with 600 μL of medium, and transmigration was allowed to proceed for 6 h at 37 °C. After incubation, inserts were washed in PBS, fixed in methanol, stained with 1% crystal violet solution (Sigma-Aldrich) for 15 min, and air-dried. Images of each insert were collected using a Nikon Eclipse80i microscope. Transmigrated ATX-KO B16-F10 cells were counted and compared to the transmigrated control WT cells. Each experiment was performed in quadruplicate. To identify any effect of ATX-producing ATII cells on B16-F10 transmigration, 5 × 10^5^ WT or ATX-KO ATII cells were plated in a 24-well plate after isolation and maintained in culture for 6 days before performing transmigration assay.

### 2.10. Cell Proliferation Assay

WT or ATX-KO B16-F10 cells were plated in a 24-well plate (2.5 × 10^3^ cells per well) in growth medium. CellTiter-Blue reagent (Promega, Madison, WI, USA) was added at the indicated time points (24 h, 48 h, 72 h, and 96 h) according to the manufacturer’s protocol and incubated for 2 h. Fluorescent signal from the CellTiter-Blue reagent was measured on a FlexStation3 plate reader (Molecular Devices) at wavelengths of 560 nm and 590 nm. Growth medium was replenished daily throughout the entire experiment.

### 2.11. Cytokine Measurement

Thirteen cytokines (IL-23, IL-1α, IFN-γ, TNF-α, MCP-1, IL-12p70, IL-1β, IL-10, IL-6, IL-27, IL-17A, IFN-β, and GM-CSF) were detected in the plasma from WT and Sftpc-KO mice, using the LEGENDPlex Mouse Inflammation Panel (Biolegend, San Diego, CA, USA), according to the manufacturer’s protocol, and quantified by flow cytometry using a ZE5 cell analyzer (Bio-Rad, Hercules, CA, USA).

### 2.12. Statistical Analysis

Data were analyzed using GraphPad Prism version 9.1.2 software (San Diego, CA, USA). The choice of statistical tests was based on normality and sample size. Where indicated, data from several independent experiments were pooled. Normality determination was performed using Shapiro–Wilk test when the n-number was superior to 6. If the sample distribution was normal, the parametric unpaired *t*-test with a two-tailed *p*-value was applied. If the n-number was too small (less than 6), a non-parametric Mann–Whitney test was used. *p*-values < 0.05 (denoted by *), <0.01 (**), <0.001 (***), and <0.0001 (****) were considered to be statistically significant.

## 3. Results

### 3.1. Generation and Validation of an Inducible Conditional KO Mice in Which ATX Is Specifically Deleted in ATII Cells

The B16-F10 murine melanoma model is a widely used model to study melanoma progression and metastasis in immunocompetent mice. When injected intravenously, B16-F10 cells readily metastasize to the lungs, allowing the study of the late events associated with metastasis. Previously, we demonstrated that shRNA-mediated silencing of ATX, a motility-promoting factor that is highly expressed in melanoma, partially reduced the number of B16-F10 metastatic nodules in the lungs [17]. Our findings suggest that tumor-derived ATX is only partially responsible for the metastatic progression of B16-F10 and that other factors may be involved in mediating metastasis. We considered the possibility that lung stromal-derived ATX may play a role in regulating the metastatic progression of B16-F10. To address our hypothesis, we used a dual approach where ATX was conditionally deleted in the lung TME’s key cell types and generation of an ATX-KO melanoma cell line.

**Figure 1:**
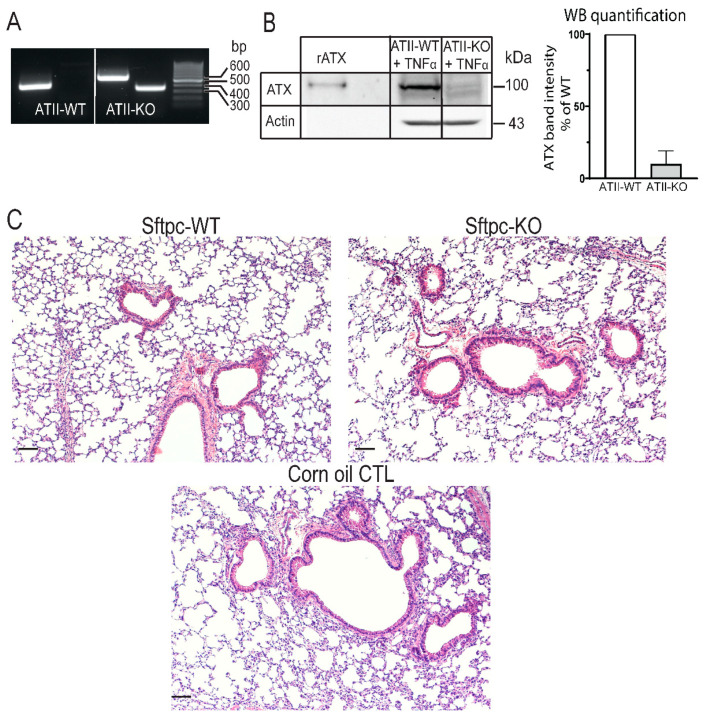
In vitro characterization of Sftpc-WT and Sftpc-KO mice. (**A**) Agarose electrophoresis of PCR-amplified Enpp2 allele of ATII cells isolated from Sftpc-WT (lanes 1 and 2) and Sftpc-KO (lanes 3 and 4) mice, treated in vivo with TAM (100 mg/kg/day for 5 days). The size of the Enpp2 WT allele is 441 bp, Enpp2 floxed allele is 540 bp, and Enpp2 deleted allele is 370 bp. The PCR product from ATII cells isolated from Sftpc-WT mouse showed a band corresponding to Enpp2 WT allele (lane 1) and no Enpp2 deleted allele was detected (lane 2). In contrast, ATII cells isolated from Sftpc-KO mouse showed a floxed allele (lane 3) and a deleted Enpp2 allele (lane 4). (**B**) WB analysis of cell lysates from ATII cells from Sftpc-WT mice (lane 2) and Sftpc-KO mice (lane 3) treated with TAM. Recombinant ATX (rATX, lane 1) was used as a positive control. Two weeks post-TAM treatment, ATII cells were isolated from Sftpc-WT and Sftpc-KO mice and put in culture for 5 days. Eighteen hours prior to lysate being harvested, cells were cultured in serum-free medium + 10 ng/mL of TNFα, in order to stimulate ATX production. One hundred fifty micrograms of protein was loaded into an 8% SDS-PAGE. A ~100 kDa band corresponding to ATX can be observed. Graph of the densitograms represents the percent of ATX band intensity normalized to the WT. ATII cells isolated from Sftpc-KO mice show a 90% decrease in band intensity (mean ± SD of 3 independent experiments). (**C**) Representative images of H&E stained 5 μm lung sections from TAM-treated naïve Sftpc-WT (**left**) and Sftpc-KO (**right**) and corn-oil-treated control (**lower** panel) mice. There was no sign of major histopathological lesion observed between the three different cohorts of lungs. Lungs were harvested two weeks post-TAM treatment, inflated with 10% formalin, fixed, and sectioned. Scale bars represent 100 μm (10× magnification).

In order to determine whether ATX derived from ATII cells modulates B16-F10 metastasis to the lung, we crossed Sftpc-CreER^T2^ mice with Enpp2^fl/fl^ mice to specifically delete the ATX gene in ATII cells (i.e., Sftpc-KO mice). We validated the KO efficiency of ATX in this model by comparing the ATX expression in isolated ATII cells after in vivo induction by TAM. Following PCR amplification, Cre-mediated deletion of the floxed ATX allele was detected only in ATII cells isolated from Sftpc-KO mice (Figure 1A). Similarly, we detected a 90% reduction in ATX protein expression by immunoblot in cells isolated from Sftpc-KO mice (Figure 1B and Figure S1) compared to those from WT littermates, after TNFα stimulation.

Because adverse effects of Cre recombinase expression and toxicity of TAM treatment have been reported in several studies [28,29,30], mice were allowed to rest for 14 days following TAM treatment prior to inoculation with B16-F10 cells. We evaluated the histopathology of lungs at the time of tumor inoculation (i.e., 14 days post-treatment), from naïve Sftpc-WT and Sftpc-KO mice, treated with 100 mg of TAM/kg/day or treated with corn oil for 5 days. No major pathological alterations in lung histology were observed regardless of the genotype (Figure 1C).

### 3.2. Generation and Characterization of ATX-KO B16-F10 Cell Line

**Figure 2:**
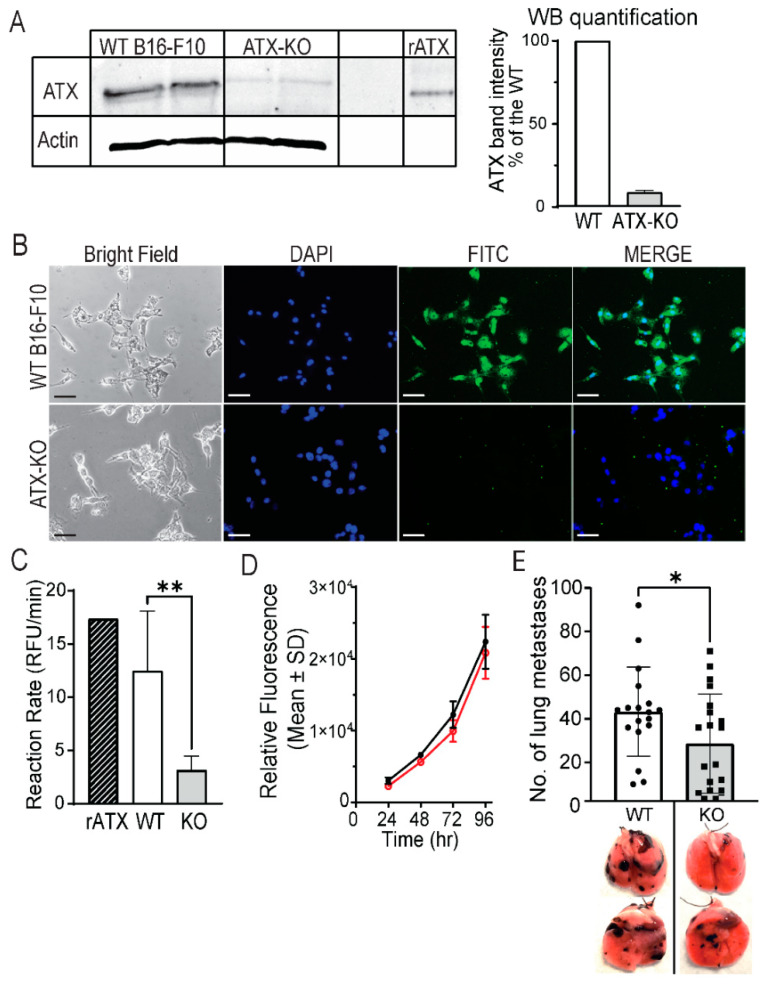
ATX derived from B16-F10 partially controls the progression of lung metastasis. (**A**) Western blot analysis of cell lysates performed in two technical repeats of WT B16-F10 cells (lanes 1 and 2, respectively) and ATX-KO B16-F10 cells (lanes 3 and 4, respectively). Recombinant ATX (rATX, lane 6) was used as a positive control. Densitometric quantification of the ATX band showed an average 91% decrease in ATX expression in ATX-KO B16-F10 cell lysate compared to WT B16-F10 cells (mean ± SD of 4 independent experiments). Cell lines were cultured for 18 h in serum-free medium before lysates were harvested. One hundred micrograms of protein was loaded into an 8% SDS-PAGE. (**B**) ATX immunofluorescence staining in WT and ATX-KO B16-F10 cells. Cells were stained for ATX (green) using the 4F1 antibody at 1:100 dilution, with DAPI nuclear counterstain (1:5000). Upper panels show the staining of WT B16-F10 cells, whereas lower panels show the staining of ATX-KO B16-F10 cells. Scale bars represent 100 μm (20× magnification). (**C**) Quantification of ATX activity in concentrated conditioned medium (CCM) from WT and ATX-KO B16-F10. Crosshatched bar represents 10 nM recombinant ATX (rATX) positive control, white bar corresponds to the ATX activity in the CCM of WT B16-F10 cells (*n* = 5), and gray bar is the activity measured in CCM of ATX-KO B16-F10 cells (*n* = 5). ATX-KO B16-F10 cells present a 74.3% decrease in ATX activity compared to WT B16-F10 cells (Mann–Whitney, ** *p* = 0.0079). (**D**) Comparison of the growth rate between WT (black) and ATX-KO (red) B16-F10 performed in six replicates; representative of three independent experiments. (**E**) Metastatic foci in the lungs of C57BL/6 mice inoculated with 1 × 10^5^ WT B16-F10 cells (white bar, *n* = 18) or ATX-KO B16-F10 cells (gray bar, *n* = 20), and representative lung pictures from this experiment (below). Mice inoculated with ATX-KO B16-F10 cells showed a 34% decrease in lung metastases (unpaired *t*-test, * *p* = 0.04, data from 2 independent experiments).

Since B16-F10 cells express high levels of ATX, we used the CRISPR-Cas9 technology to knock out ATX in B16-F10 cells, eliminating the contribution of tumor-derived ATX in lung metastasis. Sequencing analysis performed using the Synthego ICE software showed that a KO efficiency of 86% was achieved. Deletion of ATX was confirmed using WB (Figure 2A and Figure S2) and immunocytological staining (Figure 2B) for ATX protein expression. Densitometric quantification of the ATX band observed in lysates from ATX-KO B16-F10 cells showed an average 91% reduction in band intensity compared to cell lysates from non-edited WT cells. Furthermore, enzymatic activity measurement in conditioned medium generated from ATX-KO B16-F10 or WT B16-F10 cells showed a 74.3% reduction in lysophospholipase D activity (Figure 2C, Mann–Whitney, *p* = 0.0079). Of note, there was no detectable difference in cell growth between the WT and the ATX-KO B16-F10 cells (Figure 2D). Inoculation of 1 × 10^5^ ATX-KO B16-F10 cells into C57BL/6 mice significantly reduced the number of lung metastasis by 34% (unpaired *t*-test, *p* = 0.04), supporting the notion that tumor-derived ATX controls only a portion of the metastatic process.

### 3.3. Deleting ATX from ATII Cells Reduces the Number of ATX-KO B16-F10 Lung Metastases

**Figure 3:**
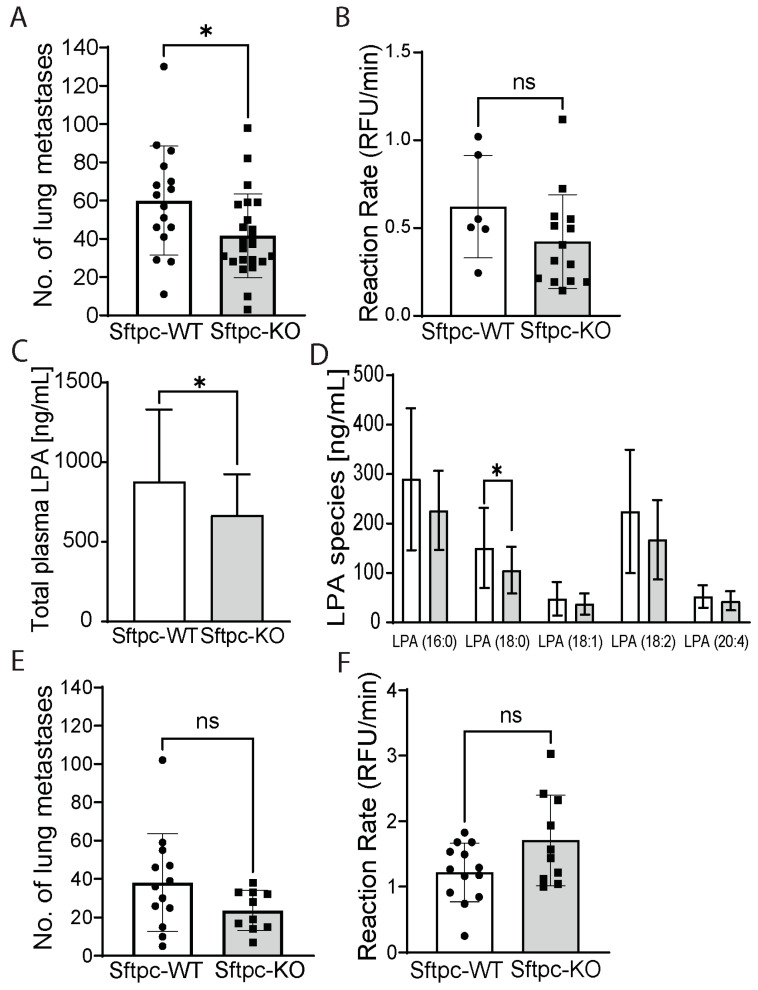
Only combined KO of ATX in B16-F10 cells and ATII cells decreases lung metastasis burden compared to KO in ATII cells alone. (**A**) Metastatic nodules in the lungs of Sftpc-WT (white bar, *n* = 16) and Sftpc-KO (gray bar, *n* = 23) mice inoculated with 1.5 × 10^5^ ATX-KO B16-F10 cells. Sftpc-KO mice showed a 30% decrease in metastatic nodules (unpaired *t*-test, * *p* = 0.028 from 2 independent experiments). (**B**) ATX activity in the BALF from Sftpc-WT (white bar, *n* = 6) and Sftpc-KO (gray bar, *n* = 14) mice inoculated with 1.5 × 10^5^ ATX-KO B16-F10 cells. There was no statistical difference between the two genotypes (unpaired *t*-test, *p* = 0.1551). (**C**) Total LPA species in the plasma of Sftpc-WT (white bar, *n* = 16) and Sftpc-KO (gray bar, *n* = 23) analyzed by mass spectrometry. Values are mean ± SD. Unpaired *t*-test, * *p* = 0.0426. (**D**) LPA species in the plasma of Sftpc-WT (white bar, *n* = 16) and Sftpc-KO (gray bar, *n* = 23) mice analyzed by mass spectrometry. Values are mean ± SD. * *p* = 0.0305, unpaired *t*-test. (**E**) Metastatic nodules in the lungs of Sftpc-WT (white bar, *n* = 13) and Sftpc-KO (gray bar, *n* = 10) mice inoculated with 1.5 × 10^5^ WT B16-F10 cells. There was no statistical difference between the two genotypes (unpaired *t*-test, *p* = 0.1063). (**F**) ATX activity in the BALF from Sftpc-WT (white bar, *n* = 13) and Sftpc-KO (gray bar, *n* = 10) mice. There was no statistical difference between the two genotypes (unpaired *t*-test, *p* = 0.052).

Injection of ATX-KO B16-F10 cells into Sftpc-KO or Sftpc-WT control littermates allowed us to then investigate the specific contribution of ATII-derived ATX in lung metastasis. We found that Sftpc-KO mice inoculated with ATX-KO B16-F10 cells showed a statistically significant 30% decrease in the number of metastatic lung nodules when compared to Sftpc-WT mice (Figure 3A, unpaired *t*-test, *p* = 0.028), suggesting that ATX derived from ATII cells could modulate the metastatic progression. Although a 30% decrease in ATX activity was also observed in the BALF of Sftpc-KO mice, no statistical difference was noted (Figure 3B, unpaired *t*-test, *p* = 0.1551). Interestingly, levels of various LPA species (16:0-, 17:0-, 18:0-, 18:1-, 18:2-, and 20:4-LPA) were in general lower in the plasma of Sftpc-KO mice compared to their WT littermates (Figure 3C, unpaired *t*-test, *p* = 0.0426), with a statistically significant decrease observed for 18:0-LPA (Figure 3D, unpaired *t*-test, *p* = 0.0305).

Next, we questioned whether deleting ATX from ATII cells alone could also impact the metastatic progression of WT B16-F10 cells. We injected 1.5 × 10^5^ WT B16-F10 cells into the tail vein of Sftpc-WT or Sftpc-KO mice. Three weeks post-inoculation, mice were euthanized; lungs were removed, inflated, and fixed in formalin; and metastatic lung nodules were counted. Although the average number of metastatic nodules appeared to be trending lower in Sftpc-KO mice, it did not reach statistical significance (Figure 3E, unpaired *t*-test, *p* = 0.1063). One possible explanation could be that the total amount of ATX present in the lungs, both from the WT B16-F10 cells which highly express ATX and from other stromal cell types, may mask or compensate for the reduction in ATX generated by ATII cells, and the latter pool alone failed to significantly impact the metastatic progression of WT B16-F10 cells. Indeed, when we measured the ATX activity in the BALF of Sftpc-WT and Sftpc-KO mice inoculated with WT B16-F10 cells, we found no statistically significant difference (Figure 3F). Taken together, our data suggest that ATX derived from ATII cells may influence metastatic progression, particularly in tumors with low ATX expression.

### 3.4. Changes in Immunological Response Associated with Deleting ATX in ATII Cells

**Figure 4:**
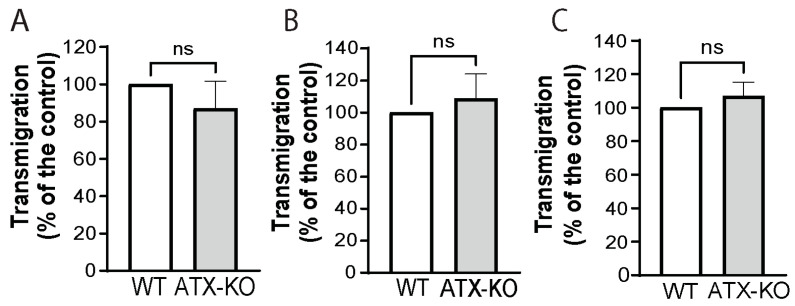
ATX derived from ATII cells does not impact the transmigration ability of B16-F10 cells. (**A**) Transmigration of WT (white bar) and ATX-KO (gray bar) B16-F10 cells after incubation in complete medium for 6 h. The experiment was performed in quadruplicate wells. No statistical difference was observed (Mann–Whitney, *p* = 0.083). (**B**) Transmigration of WT (white bar) and ATX-KO (gray bar) B16-F10 cells in the presence of ATII cells isolated from Sftpc-WT mice plated in the lower chamber. Membranes were analyzed after 6 h of incubation and performed in quadruplicate. No statistical difference was observed (Mann–Whitney, *p* = 0.404). (**C**) Transmigration of WT (white bar) and ATX-KO (gray bar) B16-F10 cells in the presence of ATII cells isolated from Sftpc-KO mice plated in the lower chamber. Membranes were analyzed after 6 h of incubation and performed in quadruplicate. No statistical difference was found (Mann–Whitney, *p* = 0.083).

To understand the mechanism(s) of the reduction in lung metastasis, we first compared the relative transmigration abilities of WT and ATX-KO B16-F10 cells in the presence of ATII cells isolated from Sftpc-WT and Sftpc-KO mice. However, no difference was observed in the transmigration of either ATX-KO B16-F10 cells compared to WT B16-F10 cells alone (Figure 4A, Mann–Whitney, *p* = 0.083) or WT and ATX-KO B16-F10 cells in presence of WT (Figure 4B, Mann–Whitney, *p* = 0.404) and ATX-KO ATII cells (Figure 4C, Mann–Whitney, *p* = 0.083).

**Figure 5:**
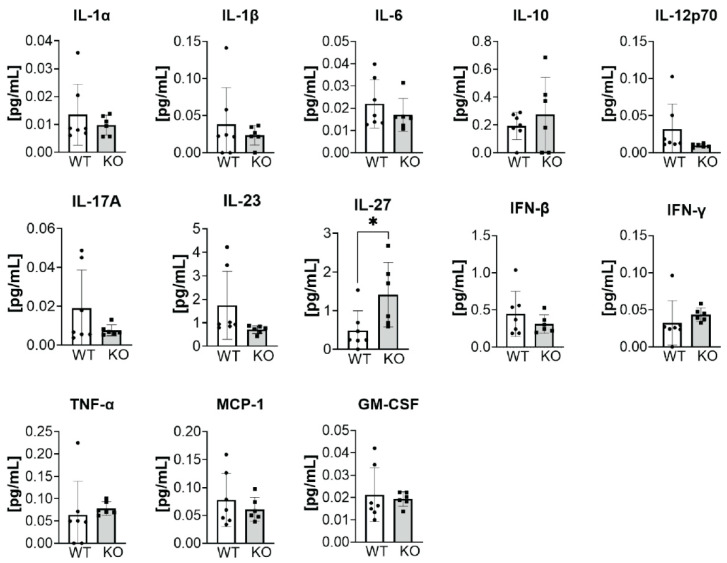
Plasma cytokine measurements in naïve Sftpc-WT and Sftpc-KO mice treated with TAM. Flow cytometry was performed to compare the basal concentration levels of 13 cytokines in the plasma of 7 Sftpc-WT (white bars) and 6 Sftpc-KO (gray bars) mice. Note that only IL-27 was different between the groups with a 3-fold higher concentration in the plasma of Sftpc-KO mice (unpaired *t*-test, * *p* = 0.032).

We previously showed that pharmacological inhibition of ATX, and consequently reduced circulating LPA levels, diminished B16-F10 metastases in mice [31]. Moreover, enhanced effector functions of CD8^+^ T cells are observed in heterozygous ATX^+/−^ mice, where systemic LPA levels are approximately halved, following immunization with a cocktail of anti-CD30, pl:C, and OVA [32]. Based on these results, we assessed the immunological status of Sftpc-WT and Sftpc-KO mice by measuring the concentrations of 13 cytokines (IL-23, IL-1α, IFN-γ, TNF-α, MCP-1, IL-12p70, IL-1β, IL-10, IL-6, IL-27, IL-17A, IFN-β, and GM-CSF) in the serum or plasma using flow cytometry and compared them across two groups consisting of (1) naïve Sftpc-WT and Sftpc-KO mice post-TAM treatment and (2) Sftpc-WT and Sftpc-KO mice inoculated with ATX-KO B16-F10 cells. The basal concentrations (i.e., post-TAM treatment and without tumor inoculation) of 12 out of the 13 cytokines examined were the same in Sftpc-WT and Sftpc-KO mice. However, a 3-fold higher level of IL-27 was found in the plasma of naïve Sftpc-KO mice compared with Sftpc-WT mice (Figure 5, unpaired *t*-test, *p* = 0.032).

**Figure 6:**
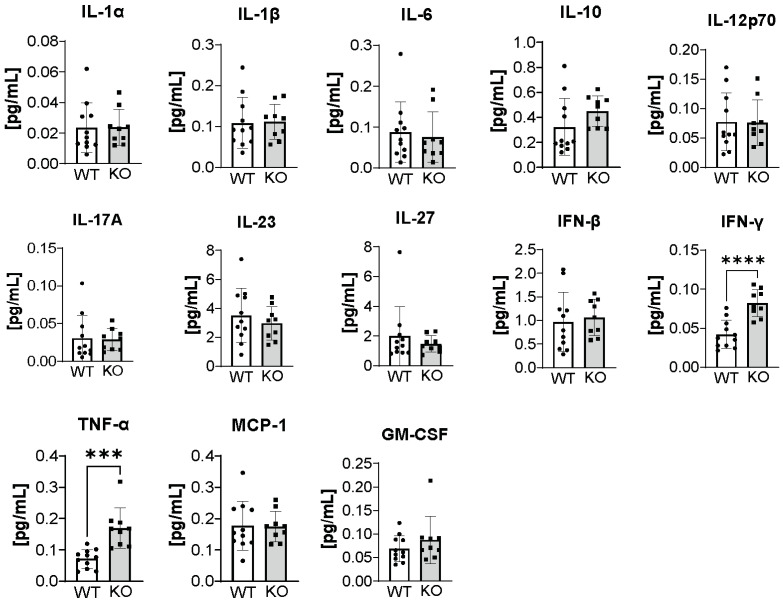
Cytokine measurements in the plasma of Sftpc-WT and Sftpc-KO mice on post-inoculation day 21. Flow cytometry was performed to compare the concentration of 13 cytokines in the plasma of Sftpc-WT (white bars) and Sftpc-KO (gray bars) mice, on day 21 post-inoculation, inoculated with 1.5 × 10^5^ ATX-KO B16-F10 cells. Sftpc-KO mice presented an increase in 2 out of the 13 cytokines (unpaired *t*-test, IFNγ, **** *p* < 0.0001; TNFα, *** *p* = 0.0003).

**Figure 7:**
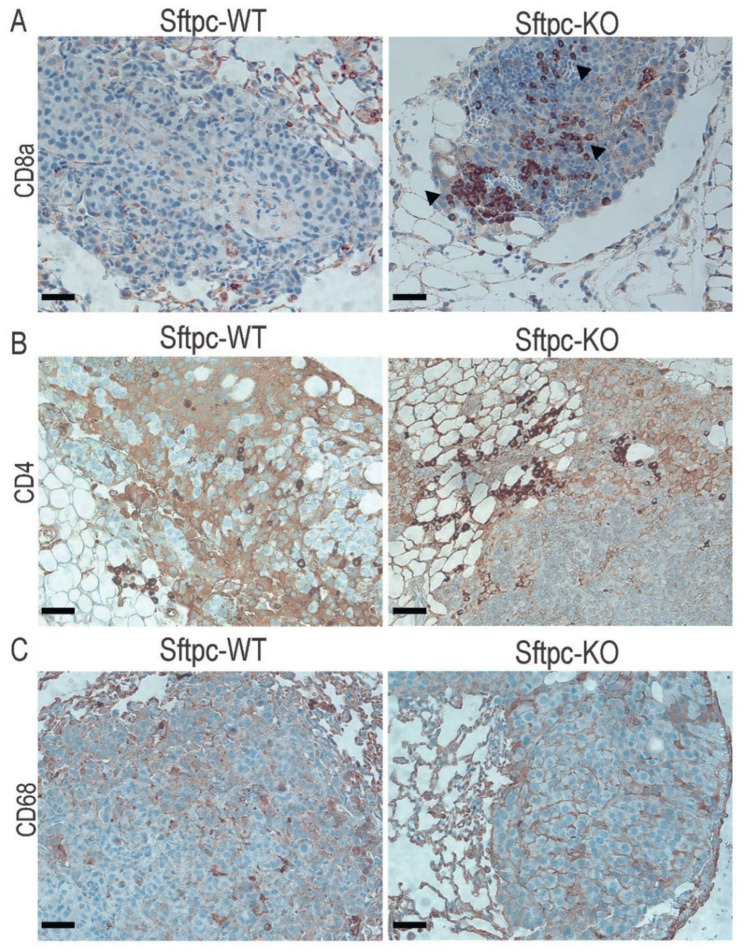
Immunostaining performed on Sftpc-WT and Sftpc-KO lung sections. Five-micrometer lung sections were stained for (**A**) CD8a, Sftpc-KO mice presented a higher CD8+ T cell infiltration (black arrows); (**B**) CD4, both groups presented nodules with sparse CD4+ infiltration; and (**C**) CD68, no infiltration of CD68^+^ cells was observed. Scale bars represent 200 μm (10× magnification).

Interestingly, when ATX-KO B16-F10 cells were inoculated into Sftpc-KO mice, we detected significant elevation of IFN-γ and TNF-α (Figure 6, unpaired *t*-test, *p* < 0.0001 and *p* = 0.0003, respectively) compared to their WT littermates on post-inoculation day 21. Because these two cytokines are mainly produced by T cells, activated macrophages, and NK cells, we examined whether Sftpc-KO mice showed enhanced infiltration of cytotoxic CD8^+^ T cells that could provide an explanation for this phenotype. Indeed, we found that Sftpc-KO mice exhibited a slightly higher number of metastatic nodules infiltrated with CD8^+^ T cells than did Sftpc-WT mice (Figure 7A). In contrast, no differences were observed in infiltration of the metastatic nodules with CD4^+^ T cells (Figure 7B) or anti-CD68^+^ macrophages (Figure 7C). These results suggest that perhaps Sftpc-KO mice inoculated with ATX-KO B16-F10 cells could better control the metastatic progression by developing a more robust adaptive immune response.

## 4. Discussion

The ATX–LPA pathway regulates several mechanisms in the progression and metastasis of different types of cancers [9,13]. We and others have shown that in addition to carcinoma cells, stromal cells in the TME are an important source of ATX and LPA. For example, we showed previously that the ATX inhibitor BMP22 significantly reduced bone metastasis of MDA-B02 cells, a breast cancer cell line that does not express ATX [14]. In this model, ATX derived from platelets was found to be partially responsible for the metastatic progression of MDA-B02 cells, suggesting that stroma-derived ATX–LPA may make an equally important contribution as tumor-derived ATX to this process. In the present study, we report on our findings on the contribution of ATX originating from ATII cells in the development of melanoma-derived lung metastasis using gene KO methods.

Although ATII cells represent a small portion of the lung stroma (~5%), they also synthesize and secrete ATX, and after lung injury and remodeling, they serve as progenitors of type I pneumocytes. In addition to these important roles, we show for the first time that deleting ATX from ATII cells led to a significant 30% decrease in ATX-KO B16-F10 lung metastases. While ATX activity in the BALF of Sftpc-KO mice appeared to trend lower, it was not statistically significant. Nonetheless, we observed a slight decrease in total plasma LPA levels, with a significant decrease in 18:0-LPA. On the contrary, deleting ATX from ATII cells alone was not sufficient to significantly decrease the number of WT B16-F10 lung metastatic nodules. We note that the Cre/loxP TAM-inducible KO system used in this study does not provide 100% efficient recombination. Furthermore, we must take into consideration that ATII cells represent a low fraction of the total lung cell population. Thus, the lack of an effect of ATX KO in ATII cells alone on reducing the metastatic burden of WT B16-F10 cells could be explained by the hypothesis that WT B16-F10 cells and possibly other stromal cells in the lung TME produce substantially more ATX and LPA than do ATII cells alone. This hypothesis could also explain why we failed to detect a statistically significant difference in ATX activity in the BALF from Sftpc-WT compared with that of Sftpc-KO mice.

We did not observe differences in the transmigration of ATX-KO B16-F10 cells towards ATII cells isolated from either Sftpc-WT or Sftpc-KO mice that could account for the reduced metastatic burden observed in Sftpc-KO mice. Therefore, our attention turned to the relative levels of antitumor immunity in Sftpc-WT versus Sftpc-KO mice. We performed immunohistological staining for CD8-, CD4-, and CD68-positive tumor-infiltrating immune cells and measured relative levels of cytokines produced in Sftpc-WT and Sftpc-KO mice. At the basal level, mice from both genotypes treated with TAM and without tumor inoculation showed no difference in 12 out of the 13 cytokines tested, with the exception being IL-27, which was increased by ~3-fold in Sftpc-KO mice. A possible explanation for this observation might be that the increase in IL-27 was caused by a natural reactive response to TAM treatment. Indeed, Bockerstett et al. demonstrated that gastric lesions caused by TAM administration can be reduced by administration of recombinant IL-27, leading to suppressed expression of proinflammatory genes [33]. However, this alone could not account for the nearly 3-fold increase in IL-27 being observed only in Sftpc-KO mice and not in Sftpc-WT mice, since both received the same TAM treatment. Therefore, we suggest that this difference might be attributed to the model itself, where the KO of ATX in ATII cells could directly or indirectly increase IL-27 production. It is also possible that the genetic recombination in the Sftpc-KO mice might delay and/or prolong IL-27 production because TAM can modulate the production of some cytokines [34]. These open questions will have to be addressed experimentally in the future.

Examination of cytokine levels in tumor-bearing mice on post-inoculation day 21 showed that inoculation of ATX-KO B16-F10 cells led to a selective increase in IFNγ and TNFα levels only in Sftpc-KO mice. Although these two cytokines play paradoxical roles in cancer [35,36], several reports have demonstrated their anticancer activities [37,38,39]. For example, IFNγ is mainly produced by CD8^+^ and CD4^+^ T cells and can directly enhance CD8^+^ T cell motility and cytotoxicity [40]. In support of this, we observed an increased recruitment of CD8^+^ T cells to the sites of metastases in Sftpc-KO mice that could be attributed to the increase in IFNγ levels. This could provide an explanation for why these mice presented decreased numbers of metastatic nodules. It is interesting to note that this notion is in agreement with our previous reports demonstrating that stromal LPA has a profound role in inhibiting tumor immunity [41] by blocking the activation of the T cell [42] and B cell receptors [43], in turn attenuating cancer surveillance and tumor cell killing. However, other reports have implicated the ATX–LPA axis in the polarization of naïve T cells [44], T cell motility [45], and increasing T cell entry to secondary lymphoid organs [44,46]. Taken together, these point to the complexity and the spatial dependence of the actions of the ATX–LPA axis.

Alternatively, the reduced number of lung metastases could also be attributed to the increased levels of IL-27 in Sftpc-KO mice following TAM treatment. Indeed, IL-27 has been shown to enhance NK cell activity in the B16-F10 model [47]. Therefore, the early overexpression of IL-27 observed at the basal level in Sftpc-KO mice might establish a faster activation of NK cells, leading to a better control of lung metastasis development. Future and extensive studies are needed to fully delineate the underlying mechanisms that will allow us to better understand how the modulation of these cytokines by the ATX–LPA axis impacts immune regulation in the TME.

## 5. Conclusions

Taken together, our data generated using cell-type-specific KO methods suggest that ATX produced by ATII cells together with ATX synthesized by B16-F10 melanoma cells can impair the adaptive antitumor immune response. They further suggest that inhibiting both sources of ATX reduces the metastatic burden by increasing cytotoxic CD8^+^ T cell infiltration. This in turn lends support to new arguments in favor of the therapeutic exploration of ATX inhibitors for the control of metastatic spread. This is a key finding as it suggests that ATX derived from ATII cells could affect the metastatic progress, particularly in tumors with low ATX expression. More importantly, it indicates that therapeutic targeting of ATX could still be a viable option even for patients whose tumors do not express high levels of ATX. This is a new finding, and an extension from our previous work using a different cancer model, where ATX derived from platelets modulates the metastatic progression of breast cancer cells that do not express ATX to the bone.

## Data Availability

The data presented in this study are available on request from the corresponding author.

## Supplementary Materials

The following are available online at https://www.mdpi.com/article/10.3390/cancers14061586/s1, Figure S1: Western blot analysis of cell lysates from ATII cells isolated from Sftpc-WT and Sftpc-KO mice, Figure S2: Western blot analysis of cell lysates from WT and ATX-KO B16-F10 cells.

## References

[1] Fares C.M., Van Allen E.M., Drake C.G., Allison J.P., Hu-Lieskovan S. (2019). Mechanisms of Resistance to Immune Checkpoint Blockade: Why Does Checkpoint Inhibitor Immunotherapy Not Work for All Patients?. Am. Soc. Clin. Oncol. Educ. Book.

[2] Michielin O., Atkins M.B., Koon H.B., Dummer R., Ascierto P.A. (2020). Evolving impact of long-term survival results on metastatic melanoma treatment. J. Immunother. Cancer.

[3] van Zijl F., Krupitza G., Mikulits W. (2011). Initial steps of metastasis: Cell invasion and endothelial transmigration. Mutat. Res. Mutat. Res..

[4] Zhuyan J., Chen M., Zhu T., Bao X., Zhen T., Xing K., Wang Q., Zhu S. (2020). Critical steps to tumor metastasis: Alterations of tumor microenvironment and extracellular matrix in the formation of pre-metastatic and metastatic niche. Cell Biosci..

[5] Brodt P. (2016). Role of the Microenvironment in Liver Metastasis: From Pre- to Prometastatic Niches. Clin. Cancer Res..

[6] Hirata E., Sahai E. (2017). Tumor Microenvironment and Differential Responses to Therapy. Cold Spring Harb. Perspect. Med..

[7] Benavente S., Sánchez-García A., Naches S., Lleonart M.E., Lorente J. (2020). Therapy-Induced Modulation of the Tumor Microenvironment: New Opportunities for Cancer Therapies. Front. Oncol..

[8] Tigyi G.J., Yue J., Norman D.D., Szabo E., Balogh A., Balazs L., Zhao G., Lee S.C. (2019). Regulation of tumor cell-Microenvironment interaction by the autotaxin-lysophosphatidic acid receptor axis. Adv. Biol. Regul..

[9] Aiello S., Casiraghi F. (2021). Lysophosphatidic Acid: Promoter of Cancer Progression and of Tumor Microenvironment Development. A Promising Target for Anticancer Therapies?. Cells.

[10] Stracke M.L., Krutzsch H.C., Unsworth E.J., Arestad A., Cioce V., Schiffmann E., Liotta L.A. (1992). Identification, purification, and partial sequence analysis of autotaxin, a novel motility-stimulating protein. J. Biol. Chem..

[11] Benesch M.G.K., Tang X., Dewald J., Dong W.-F., Mackey J.R., Hemmings D.G., McMullen T.P.W., Brindley D.N. (2015). Tumor-induced inflammation in mammary adipose tissue stimulates a vicious cycle of autotaxin expression and breast cancer progression. FASEB J..

[12] Brindley D.N., Lin F.-T., Tigyi G.J. (2013). Role of the autotaxin–lysophosphatidate axis in cancer resistance to chemotherapy and radiotherapy. Biochim. Biophys. Acta (BBA)-Mol. Cell Biol. Lipids.

[13] Zhang X., Li M., Yin N., Zhang J. (2021). The Expression Regulation and Biological Function of Autotaxin. Cells.

[14] Leblanc R., Lee S.-C., David M., Bordet J.-C., Norman D.D., Patil R., Miller D., Sahay D., Ribeiro J., Clézardin P. (2014). Interaction of platelet-derived autotaxin with tumor integrin αVβ3 controls metastasis of breast cancer cells to bone. Blood.

[15] Auciello F.R., Bulusu V., Oon C., Tait-Mulder J., Berry M., Bhattacharyya S., Tumanov S., Allen-Petersen B.L., Link J., Kendsersky N.D. (2019). A Stromal Lysolipid–Autotaxin Signaling Axis Promotes Pancreatic Tumor Progression. Cancer Discov..

[16] Magkrioti C., Oikonomou N., Kaffe E., Mouratis M.-A., Xylourgidis N., Barbayianni I., Megadoukas P., Harokopos V., Valavanis C., Chun J. (2018). The autotaxin-lysophosphatidic acid axis promotes lung carcinogenesis. Cancer Res..

[17] Gotoh M., Fujiwara Y., Yue J., Liu J., Lee S., Fells J., Uchiyama A., Murakami-Murofushi K., Kennel S., Wall J. (2012). Controlling cancer through the autotaxin-lysophosphatidic acid receptor axis. Biochem. Soc. Trans..

[18] Lee S.-C., Fujiwara Y., Liu J., Yue J., Shimizu Y., Norman D.D., Wang Y., Tsukahara R., Szabo E., Patil R. (2015). Autotaxin and LPA1 and LPA5 Receptors Exert Disparate Functions in Tumor Cells versus the Host Tissue Microenvironment in Melanoma Invasion and Metastasis. Mol. Cancer Res..

[19] Minn A.J., Gupta G.P., Siegel P.M., Bos P.D., Shu W., Giri D.D., Viale A., Olshen A.B., Gerald W.L., Massagué J. (2005). Genes that mediate breast cancer metastasis to lung. Nature.

[20] Ruaro B., Salton F., Braga L., Wade B., Confalonieri P., Volpe M.C., Baratella E., Maiocchi S., Confalonieri M. (2021). The History and Mystery of Alveolar Epithelial Type II Cells: Focus on Their Physiologic and Pathologic Role in Lung. Int. J. Mol. Sci..

[21] Castranova V., Rabovsky J., Tucker J., Miles P. (1988). The alveolar type II epithelial cell: A multifunctional pneumocyte. Toxicol. Appl. Pharmacol..

[22] Zhao J., He D., Berdyshev E., Zhong M., Salgia R., Morris A.J., Smyth S.S., Natarajan V., Zhao Y. (2011). Autotaxin induces lung epithelial cell migration through lysoPLD activity-dependent and -independent pathways. Biochem. J..

[23] Oikonomou N., Mouratis M.-A., Tzouvelekis A., Kaffe E., Valavanis C., Vilaras G., Karameris A., Prestwich G.D., Bouros D., Aidinis V. (2012). Pulmonary Autotaxin Expression Contributes to the Pathogenesis of Pulmonary Fibrosis. Am. J. Respir. Cell Mol. Biol..

[24] Sinha M., Lowell C. (2016). Isolation of Highly Pure Primary Mouse Alveolar Epithelial Type II Cells by Flow Cytometric Cell Sorting. Bio-Protocol.

[25] Sun F., Xiao G., Qu Z. (2017). Isolation of Murine Alveolar Type II Epithelial Cells. Bio-Protocol.

[26] Van Meeteren L.A., Ruurs P., Stortelers C., Bouwman P., van Rooijen M.A., Pradère J.P., Pettit T.R., Wakelam M.J.O., Saulnier-Blache J.S., Mummery C.L. (2006). Autotaxin, a Secreted Lysophospholipase D, Is Essential for Blood Vessel Formation during Development. Mol. Cell. Biol..

[27] Okudaira M., Inoue A., Shuto A., Nakanaga K., Kano K., Makide K., Saigusa D., Tomioka Y., Aoki J. (2014). Separation and quantification of 2-acyl-1-lysophospholipids and 1-acyl-2-lysophospholipids in biological samples by LC-MS/MS. J. Lipid Res..

[28] Huh W.J., Khurana S.S., Geahlen J.H., Kohli K., Waller R.A., Mills J.C. (2012). Tamoxifen Induces Rapid, Reversible Atrophy, and Metaplasia in Mouse Stomach. Gastroenterology.

[29] Etori S., Nakano R., Kamada H., Hosokawa K., Takeda S., Fukuhara M., Kenmotsu Y., Ishimine A., Sato K. (2017). Tamoxifen-induced Lung Injury. Intern. Med..

[30] Donocoff R.S., Teteloshvili N., Chung H., Shoulson R., Creusot R.J. (2020). Optimization of tamoxifen-induced Cre activity and its effect on immune cell populations. Sci. Rep..

[31] Gupte R., Patil R., Liu J., Wang Y., Lee S.C., Fujiwara Y., Fells J., Bolen A.L., Emmons-Thompson K., Yates C.R. (2011). Benzyl and Naphthalene Methylphosphonic Acid Inhibitors of Autotaxin with Anti-invasive and Anti-metastatic Activity. ChemMedChem.

[32] Mathew D., Kremer K.N., Strauch P., Tigyi G., Pelanda R., Torres R.M. (2019). LPA5 Is an Inhibitory Receptor That Suppresses CD8 T-Cell Cytotoxic Function via Disruption of Early TCR Signaling. Front. Immunol..

[33] Bockerstett K.A., Petersen C.P., Noto C.N., Kuehm L.M., Wong C.F., Ford E.L., Teague R.M., Mills J.C., Goldenring J.R., DiPaolo R.J. (2020). Interleukin 27 Protects From Gastric Atrophy and Metaplasia During Chronic Autoimmune Gastritis. Cell. Mol. Gastroenterol. Hepatol..

[34] Behjati S., Frank M. (2009). The Effects of Tamoxifen on Immunity. Curr. Med. Chem..

[35] Wang X., Lin Y. (2008). Tumor necrosis factor and cancer, buddies or foes?. Acta Pharmacol. Sin..

[36] Jorgovanovic D., Song M., Wang L., Zhang Y. (2020). Roles of IFN-γ in tumor progression and regression: A review. Biomark. Res..

[37] Fenton S., Saleiro D., Platanias L. (2021). Type I and II Interferons in the Anti-Tumor Immune Response. Cancers.

[38] Burke J.D., Young H.A. (2019). IFN-γ: A cytokine at the right time, is in the right place. Semin. Immunol..

[39] Montfort A., Colacios C., Levade T., Andrieu-Abadie N., Meyer N., Ségui B. (2019). The TNF Paradox in Cancer Progression and Immunotherapy. Front. Immunol..

[40] Bhat P., Leggatt G., Waterhouse N., Frazer I. (2017). Interferon-γ derived from cytotoxic lymphocytes directly enhances their motility and cytotoxicity. Cell Death Dis..

[41] Lee S.C., Dacheux M.A., Norman D.D., Balázs L., Torres R.M., Augelli-Szafran C.E., Tigyi G.J. (2020). Regulation of Tumor Immunity by Lysophosphatidic Acid. Cancers.

[42] Oda S.K., Strauch P., Fujiwara Y., Al-Shami A., Oravecz T., Tigyi G., Pelanda R., Torres R.M. (2013). Lysophosphatidic Acid Inhibits CD8 T-cell Activation and Control of Tumor Progression. Cancer Immunol. Res..

[43] Hu J., Oda S.K., Shotts K., Donovan E.E., Strauch P., Pujanauski L.M., Victorino F., Al-Shami A., Fujiwara Y., Tigyi G. (2014). Lysophosphatidic Acid Receptor 5 Inhibits B Cell Antigen Receptor Signaling and Antibody Response. J. Immunol..

[44] Zhang Y., Chen Y.-C.M., Krummel M.F., Rosen S.D. (2012). Autotaxin through Lysophosphatidic Acid Stimulates Polarization, Motility, and Transendothelial Migration of Naive T Cells. J. Immunol..

[45] Knowlden S.A., Capece T., Popovic M., Chapman T., Rezaee F., Kim M., Georas S.N. (2014). Regulation of T Cell Motility In Vitro and In Vivo by LPA and LPA2. PLoS ONE.

[46] Kanda H., Newton R., Klein R., Morita Y., Gunn M.D., Rosen S.D. (2008). Autotaxin, an ectoenzyme that produces lysophosphatidic acid, promotes the entry of lymphocytes into secondary lymphoid organs. Nat. Immunol..

[47] Liu Z., Yu J., Carson W.E., Bai X.F. (2013). The role of IL-27 in the induction of anti-tumor cytotoxic T lymphocyte response. Am. J. Transl. Res..

