# Supplementary material for: Prometastatic Effect of ATX Derived from Alveolar Type II Pneumocytes and B16-F10 Melanoma Cells
Source: Cancers (Basel). 2022 Mar 21;14(6):1586. doi: 10.3390/cancers14061586 (PMC8946623; doi:10.3390/cancers14061586)
Supplement: Supplementary file 1 [file cancers-14-01586-s001.zip › cancers-1593317-SI.pdf]

Supplementary Materials:

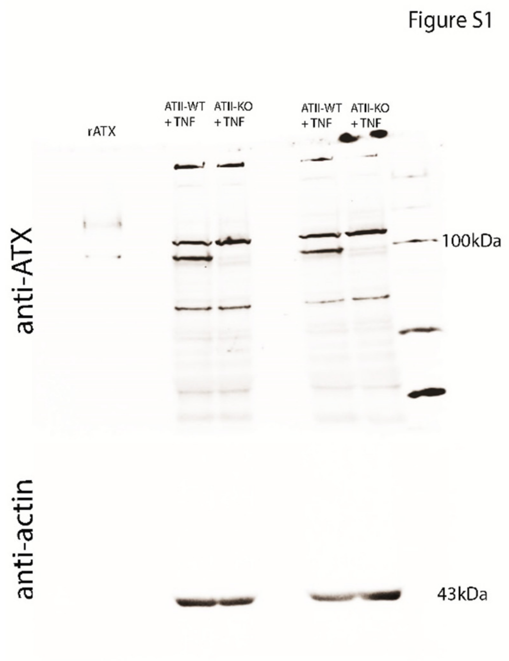

Figure S1. Western blot analysis of cell lysates from ATII cells from Sftpc-WT mice (lanes 3 and 6) and Sftpc-KO mice (lanes 4 and 7) treated with TAM. Recombinant ATX (rATX, lane 1) was used as a positive control. Two weeks post-TAM treatment, ATII cells were isolated from Sftpc-WT and Sftpc-KO mice and put in culture for 5 days. Eighteen hours prior to lysate being harvested, cells were cultured in serum-free medium + 10 ng/mL of TNFα, in order to stimulate ATX production. One hundred fifty micrograms of protein was loaded into an 8% SDS-PAGE. A ~100 kDa band corresponding to ATX can be observed.

Figure S2

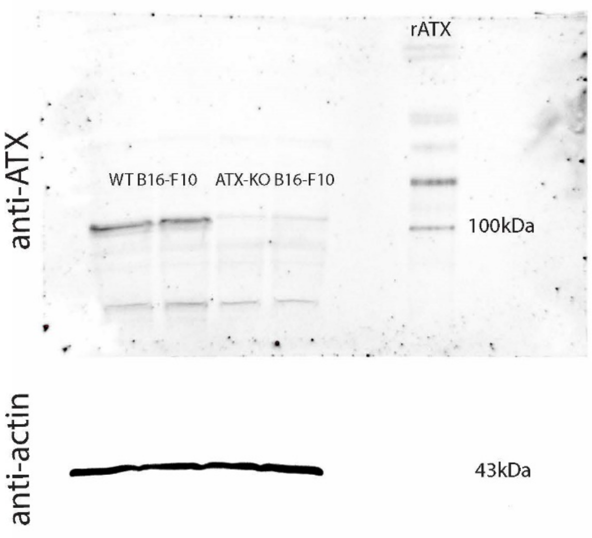

Figure S2. Western blot analysis of cell lysates performed in two technical repeats of WT B16-F10 cells (lanes 1 and 2, respectively) and ATX-KO B16-F10 cells (lanes 3 and 4, respectively). Recombinant ATX (rATX, lane 6) was used as a positive control. Cell lines were cultured for 18 h in serum-free medium before lysates were harvested. One hundred micrograms of protein was loaded into an 8% SDS-PAGE.
